# Systems biology and experimental validation indicate DDIT4, FOXO1, and STAT3 as shared key genes linking osteoporosis and sarcopenia

**DOI:** 10.3389/fgene.2025.1630705

**Published:** 2025-11-06

**Authors:** Wenjing Li, Yuechen Xing, Lihong Jiang, Jia Meng, Yue Wang, Renjie Tan, Yina Zhang

**Affiliations:** 1 Department of Geriatrics, The Second Affiliated Hospital of Harbin Medical University, Harbin, Heilongjiang, China; 2 Department of General Practice, The Second Affiliated Hospital of Harbin Medical University, Harbin, Heilongjiang, China; 3 Department of Occupational Health, School of Public Health, Harbin Medical University, Harbin, Heilongjiang, China; 4 School of Interdisciplinary Medicine and Engineering, Harbin Medical University, Harbin, Heilongjiang, China

**Keywords:** osteoporosis, sarcopenia, systems biology, disease biomarkers, machine learning, experimental validation

## Abstract

**Background:**

With the aging population, osteoporosis and sarcopenia have emerged as two prevalent age-related degenerative diseases that pose significant public health challenges. Although clinical studies increasingly report the co-occurrence of these conditions, the underlying molecular mechanisms linking them remain poorly understood.

**Methods:**

We adopted a systems biology approach to identify key biomarkers and explore their molecular roles in the interplay between osteoporosis and sarcopenia. Transcriptomic datasets were systematically analyzed to identify candidate genes. The expression patterns of core biomarkers were validated using independent datasets and *in vitro* cellular models of both diseases. Furthermore, a machine learning–based diagnostic framework was constructed using the identified biomarkers, and model interpretability was enhanced using Shapley Additive Explanations (SHAP).

**Results:**

We identified DDIT4, FOXO1, and STAT3 as three central biomarkers that play pivotal roles in the pathogenesis of both osteoporosis and sarcopenia. Their expression patterns were consistently validated across multiple independent transcriptomic datasets, and their differential expression was further confirmed using quantitative reverse transcription polymerase chain reaction (RT-PCR) in disease-relevant cellular models. A diagnostic model constructed based on biomarker genes achieved high classification accuracy across diverse validation cohorts. Moreover, SHAP analysis quantified the individual contribution of each biomarker to the model’s predictive performance.

**Conclusion:**

This study uncovers key molecular links between osteoporosis and sarcopenia, highlighting DDIT4, FOXO1, and STAT3 as shared biomarkers. The findings provide novel insights into their common pathophysiology and lay the groundwork for developing more accurate diagnostic tools and targeted therapeutic strategies.

## 1 Introduction

With continuous social progress and improvements in healthcare, human life expectancy has been steadily increasing, making population aging a global trend. The prevalence of age-related diseases increases exponentially with age (Belikov, 2019). Over 50% of diseases have been identified as contributing to the global burden in adults due to aging (Chang et al., 2019). Age-related diseases have become a significant burden on human health; among them, osteoporosis and sarcopenia are increasingly common and concerning conditions (Guo et al., 2022; Yang et al., 2023). Osteoporosis is a common systemic skeletal disorder characterized by low bone mass and an increased propensity to fracture (Compston et al., 2019). Osteoporosis is influenced by both environmental and genetic factors, with genetic factors accounting for 50%–85% of the variability (Compston et al., 2019). Genome-wide association studies have identified approximately 100 genomic loci associated with bone density and other related phenotypes (Trajanoska and Rivadeneira, 2019). For example, EN1 has been reported as a determinant of bone density and fracture risk (Zheng et al., 2015). However, the causal mechanisms for many of these associations remain unclear (Boudin and Van Hul, 2017). Sarcopenia is defined as a progressive and generalized skeletal muscle disorder involving accelerated loss of muscle mass and function (Cruz-Jentoft and Sayer, 2019). The causes of sarcopenia can vary and include changes in hormones and growth factors, imbalances in protein metabolism, and inflammation. However, the mechanisms and pathways involved are still not fully understood. Additionally, there is no consensus on the diagnostic criteria for sarcopenia (Bruyère et al., 2016; Cruz-Jentoft and Sayer, 2019; Evans et al., 2024).

Notably, the coexistence of osteoporosis and sarcopenia in the elderly population is becoming increasingly common (Kirk et al., 2022; Laskou et al., 2022; Yang et al., 2023). Muscles and bones are not merely connected physically; they are closely related at both the physiological and pathological levels (Gielen et al., 2023; Kun et al., 2023). Some even suggest that osteoporosis and sarcopenia represent two manifestations of a single disease in different physiological systems (Gielen et al., 2023; Chen et al., 2024b). Recently, Liu et al. reported the involvement of STAT3 in both postmenopausal osteoporosis and sarcopenia (Liu et al., 2024). However, current research on the relationship between these two conditions remains limited (Yang et al., 2023), and there is insufficient evidence to conclusively establish molecular links between them. Furthermore, our understanding of this relationship is still incomplete. The precise diagnosis of osteoporosis and sarcopenia is particularly challenging given the lack of standardized diagnostic criteria for sarcopenia. Additionally, since identifying disease-specific biomarkers is essential for understanding diseases and the corresponding treatments (Byron et al., 2016; Wang et al., 2022; Mahmoodi Chalbatani et al., 2023), there is a need for an accurate approach to detect molecular biomarkers of osteoporosis and sarcopenia. To address this issue, our study incorporated gene expression microarray datasets from both osteoporosis and sarcopenia patients to conduct a systems biology analysis, aiming to identify the biomarkers and uncover the molecular mechanisms connecting the two diseases. Furthermore, inspired by the success of previous related studies (Liu et al., 2014; Lipman et al., 2022; Shu et al., 2022), we developed a machine learning framework combined with computational and experimental validation to enhance the accurate identification of osteoporosis and sarcopenia.

## 2 Materials and methods

### 2.1 Overview

**FIGURE 1:**
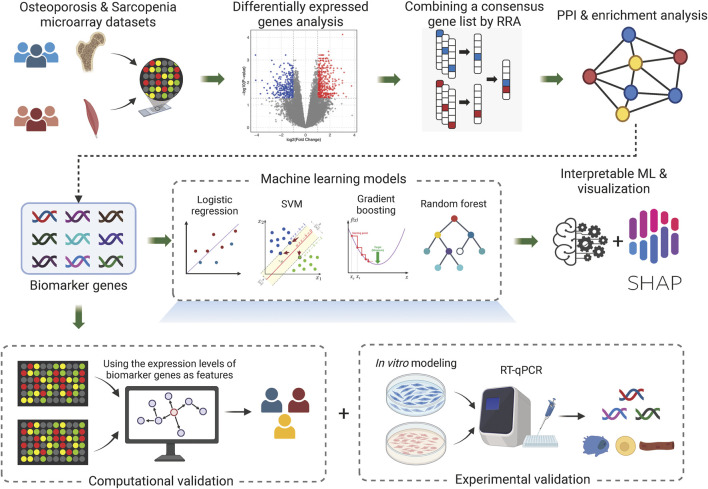
Overall workflow. The diagram represents the core workflow implemented in this study. Created in BioRender. li, w. (2025) https://BioRender.com/3uhunut.

Figure 1 illustrates the overall workflow of this study. First, we analyzed multiple microarray datasets from osteoporosis and sarcopenia studies and conducted differentially expressed genes analysis for each individual dataset. Next, we employed a robust integration approach to rank the differentially expressed genes (DEGs) across different datasets within each disease. By combining data from multiple studies, we identified common DEGs shared between osteoporosis and sarcopenia. Subsequent network and enrichment analyses identified key biomarker genes that may play pivotal roles in both diseases, shedding light on their shared molecular mechanisms. Furthermore, leveraging these biomarker genes, we developed a machine learning framework to predict the presence of osteoporosis and sarcopenia. Finally, through computational analysis, *in vitro* modeling, and RT-qPCR validation, we confirmed the robustness of our findings, underscoring the potential of the identified genes as diagnostic biomarkers and therapeutic targets for these interrelated conditions.

### 2.2 Microarray datasets and data preprocessing

We downloaded osteoporosis and sarcopenia microarray datasets from the National Institutes of Health Gene Expression Omnibus (GEO) data repository (Barrett et al., 2013; Xu et al., 2023). ‘Osteoporosis’ and ‘Sarcopenia’ were used as keywords to query relevant datasets. We limited the entry type to ‘Series’, study type to ‘Expression profiling by array’, and top organisms to ‘*Homo sapiens*’ to accurately locate the datasets required for this study. We subsequently manually reviewed the selected datasets and excluded those involving non-target traits, non-transcriptomic platforms, or redundancy across datasets. This step ensured that the final dataset collection met the specific data requirements of our study. After identifying the appropriate microarray datasets, we downloaded the expression profile information, phenotypic data, and metadata using the ‘GEOquery’ R package (Sean and Meltzer, 2007). We then used the ‘AnnoProbe’ package to annotate the probe information in the expression profiles with gene names.

To minimize batch effects and ensure the reliability of the results, we first calculated the quantiles of the gene expression set at specified levels and assessed certain statistical properties to determine whether log transformation was necessary. This transformation helps stabilize the variance and makes the data more normally distributed. We subsequently used the ‘normalizeBetweenArrays’ function from the Limma package (Ritchie et al., 2015) to normalize the expression data. For genes with multiple corresponding probes, we calculated the average expression value of these probes as the overall gene expression level. Based on the phenotypic data, we classified the samples in each dataset into case and control groups. Samples that were diagnosed with neither osteoporosis nor sarcopenia, as well as those with ambiguous phenotypic data, were classified as ‘unknown’ and excluded from downstream analyses. To further assess data consistency, we performed principal component analysis (PCA) both within each individual dataset and on the merged dataset used for machine learning model training. Any significant outliers indicating heterogeneity would be removed from downstream analysis.

### 2.3 Differential expressed genes identification

We used the Limma package in R to conduct differential expression analysis for each microarray dataset. Limma fits linear models to the expression data based on case–control phenotypes and applies empirical Bayes moderation to estimate gene-wise variances. For each gene, it computes the log fold change (logFC), raw p-value, and adjusted p-value to account for multiple testing. Although DEGs in each dataset could be typically selected using default thresholds (e.g., adjusted p-value <0.05 and $\left|\text{logFC}\right|\geq 1$ ), our study required integration across multiple datasets, which introduces challenges such as batch effects and incomplete or inconsistent gene rankings. Therefore, instead of relying solely on conventional cutoffs, we employed the robust rank aggregation (RRA) method (Kolde et al., 2012), which evaluates the ranking of genes by their logFC or p-value across different datasets to identify genes that consistently rank highly in all datasets. We used this method to combine multiple ranked lists into a single consensus gene list and set the RRA p-value <0.05 as the significance cutoff when defining the DEGs for downstream analysis.

### 2.4 Protein–protein interaction analysis and hub gene selection

To systematically elucidate the pathogenesis of osteoporosis and sarcopenia from a systems biology perspective and further explore the potential connections between the two diseases, we conducted a protein–protein interaction (PPI) analysis on the significantly ranked genes obtained from the RRA analysis results. We used the STRING (Szklarczyk et al., 2023) online database to analyze the protein interactions of these significantly ranked genes. We selected a minimum required interaction score of 0.4 (medium confidence) as the threshold to select interacting proteins. Disconnected proteins in the network were ignored, resulting in the final PPI network. We then utilized the cytoHubba tool in Cytoscape to analyze the resulting PPI network (Chin et al., 2014). To identify high-confidence key hub genes, which may serve as potential biomarker genes for molecular diagnosis, we selected the recommended MCC method along with two local-based methods (DMNC and Degree) and two global-based methods (Closeness and Betweenness). Finally, following the approach used in similar studies (Lv et al., 2022; Zhou et al., 2024), we employed Venn diagram analysis to identify high-confidence hub genes from the detected hub nodes.

### 2.5 Functional enrichment analysis

Functional enrichment analysis is a powerful tool for identifying and annotating the biological processes, molecular functions, cellular components, and signaling pathways associated with a given set of genes. We performed Kyoto Encyclopedia of Genes and Genomes (KEGG) pathway analysis and gene ontology (GO) enrichment using the ‘clusterProfiler’ (Yu et al., 2012) R package on our selected gene sets. Additionally, we conducted comprehensive functional enrichment analysis using Metascape (Zhou et al., 2019) to further interpret the selected gene sets.

### 2.6 Machine learning model and its explainable visualization

We developed a machine learning-based framework to identify and validate molecular biomarkers for osteoporosis and sarcopenia. Using the selected hub genes as feature genes, we implemented machine learning models using Scikit-learn (version 1.6.1), a widely used Python library. We experimented with four machine learning models, including logistic regression, support vector machine (SVM), gradient boosting, and random forest. All models were trained using default parameter settings provided by Scikit-learn. Specifically, logistic regression was implemented with L2 regularization ( $penalty{=}^{\prime }l2^{\prime }$ ) using the ‘lbfgs’ optimizer. The SVM model utilized a radial basis function (RBF) kernel with a regularization parameter of C=1.0 . The GradientBoostingClassifier applied a learning rate of 0.1 and 100 estimators to achieve a balance between model complexity and generalization. The RandomForestClassifier was configured with 100 trees ( n_estimators=100 ) and used $\max\_features{=}^{\prime }sqrt^{\prime }$ , a commonly adopted strategy for classification tasks.

We trained the models using microarray expression profile datasets from samples with confirmed osteoporosis and sarcopenia diagnoses. We applied normalization before integrating multiple datasets for both diseases to ensure consistency across datasets. We randomly shuffled and split the data into training (80%) and validation (20%) sets to train and validate the models, respectively.

To further analyze the internal decision-making mechanisms of the machine learning model and to provide insights into the roles these biomarker genes play in precision diagnosis, we employed Shapley additive explanations (SHAP) for model explainability. SHAP is an interpretability framework based on game theory, which calculates SHAP values by fairly distributing the model’s output among features using all possible feature combinations (Lundberg and Lee, 2017). We used the beeswarm summary plot to offer a global overview of feature importance across all predictions in the dataset.

### 2.7 Model performance and computational validation

To objectively evaluate the model’s performance, we obtained additional independent test sets. The refined model was evaluated on both the testing set and the independent datasets. Specifically, we used the refined model to predict the probabilities that each sample belonged to disease or control group. The state corresponding to the maximum probability value was selected as the predicted label. We classified a sample in the testing set as a true positive if its predicted label matched the corresponding true label. Cases without a matching predicted label were counted as false negatives, whereas predicted cases without a matching true label were counted as false positives. The performance of our model was evaluated using the receiver operating characteristic (ROC) curves and the areas under the curve (AUC).

In addition to validating the key genes using ROC curves, we analyzed the expression levels of the hub genes on the independent validation datasets. We compared the expression levels of these hub genes between the case and control samples and performed a *t*-test to statistically assess their differential expression.

### 2.8 *In vitro* modeling

To establish *in vitro* models for osteoporosis and sarcopenia, we used the mouse pre-osteoblastic cell line MC3T3-E1 and the mouse myoblast cell line C2C12 (both from ServiceBio, China). MC3T3-E1 cells were cultured in α-MEM supplemented with 10% fetal bovine serum (FBS) and 1% penicillin–streptomycin at 37 °C with 5% CO_2_. Upon reaching 70% confluence, cells were passaged using 0.25% trypsin, and passages 2 to 4 were used for experiments. To simulate osteoporotic conditions, cells were treated with dexamethasone at concentrations of 0, 0.1, 1, 10, 20, 40, 80, 160, and 320 μg/mL for 24 h. Cell viability was evaluated using the CCK-8 assay, and absorbance was measured at 450 nm. The optimal treatment condition was determined to be 10 μg/mL dexamethasone for 24 h. To verify model induction, quantitative RT-PCR was performed to assess the expression levels of osteogenic markers RUNX2 and osteoprotegerin (OPG), as well as the osteoclastogenesis-related factor RANKL (Liu et al., 2021).

Similarly, C2C12 myoblast cells were cultured in DMEM supplemented with 10% heat-inactivated FBS and 1% antibiotics under standard conditions. Upon reaching 70%–80% confluence, differentiation was induced by medium replacement, and cells were subsequently treated with dexamethasone at concentrations of 0, 3.125, 6.25, 12.5, 25, 50, 100, 200, and 400 μg/mL for 24 h. Cell viability was assessed by adding 10 μL of CCK-8 reagent per well, followed by incubation at 37 °C in the dark for 2.5 h, and absorbance was measured at 450 nm. Based on the dose-response curve, 12.5 μg/mL dexamethasone for 24 h was selected as the optimal treatment. Quantitative RT-PCR was then conducted to evaluate the expression of muscle atrophy markers Atrogin-1 and MuRF1, and the myogenic differentiation marker myogenin (MYOG), to confirm successful model establishment (Li et al., 2021; Kang et al., 2024).

### 2.9 Experimental validation

Following model establishment, we performed reverse transcription quantitative polymerase chain reaction (RT-qPCR) to validate the expression levels of identified biomarker genes in the induced osteoporosis and sarcopenia models, as well as their respective matched controls. The RT-qPCR primers targeting the coding regions of these genes were designed and synthesized by GenePharma (Shanghai, China). Total RNA was extracted using RNA extraction reagent (FastPure® Cell/Tissue Total RNA Isolation Kit V2, RC112). In the RT-qPCR reaction, the total volume was 20 μL, including 10 µL of 2× Universal SYBR Green Fast qPCR Mix (ABclonal), 0.8 µL of 10 nM primers, and 2 µL of diluted cDNA. All reactions were performed in triplicate under the following conditions: initial denaturation at 95 °C for 3 min, followed by 40 cycles of 95 °C for 5 s and 60 °C for 30s. The relative gene expression levels were normalized and analyzed using the 2^−ΔΔCt^ method.

## 3 Results

### 3.1 Data processing and differentially expressed genes selection

To identify biologically relevant differences in gene expression between disease and control groups, we aimed to identify DEGs and calculate corresponding p-value and logFC values. For this purpose, we sought to obtain a sufficient number of high-quality datasets to enable an in-depth investigation of the relationship between osteoporosis and sarcopenia. After searching public data repositories, we found that microarray datasets were more abundant, and these datasets had been validated by recent studies (Chen et al., 2022; Lv et al., 2022; Zhou et al., 2022; Chen et al., 2024a; Chen et al., 2024b; Liu et al., 2024). Consequently, we collected ten microarray gene expression datasets comprising a total of 356 samples (including 191 cases and 165 controls) from osteoporosis and sarcopenia cohorts (Supplementary Table S1). To the best of our knowledge, this study represents the largest integrated dataset to date for the co-disease analysis of osteoporosis and sarcopenia.

We next conducted PCA to assess data homogeneity (Supplementary Figure S1) and applied quality control procedures. During this process, we excluded samples associated with other diseases (e.g., from the GSE136344 dataset) to minimize potential bias and confounding effects. Subsequently, we independently normalized each dataset and performed differential expression analysis using the Limma package with default parameters to calculate p-values and logFC values.

To integrate the DEGs information across multiple datasets for each disease, we applied the RRA method. Notably, the RRA method aggregates DEGs rather than raw gene expression profiles. This approach effectively reduces batch effects and mitigates confounding issues arising from dataset integration, ensuring an unbiased analysis. As a result, we identified a total of 1,540 significantly ranked genes across five datasets for osteoporosis and 801 significantly ranked genes across two datasets for sarcopenia. Finally, we found that 99 significantly ranked genes were common between the osteoporosis and sarcopenia datasets. These 99 intersecting genes were then selected for downstream analysis.

**FIGURE 2:**
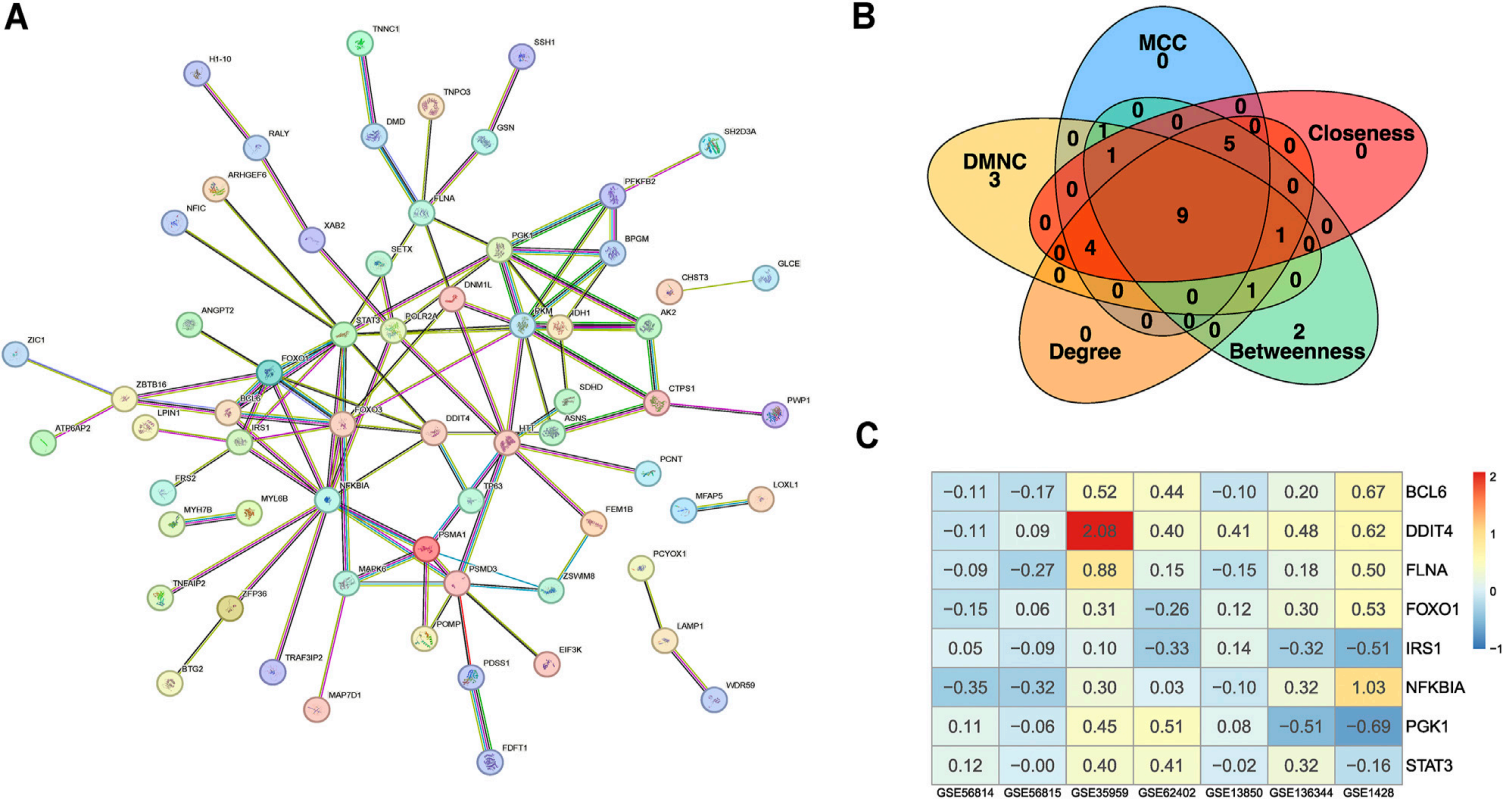
Data processing and identification of consistent biomarker genes. **(A)** PPI network constructed based on the intersecting DEGs from the integrated osteoporosis and sarcopenia datasets. Nodes represent genes, and edges represent experimentally supported interactions. **(B)** Candidate biomarker genes (BCL6, DDIT4, FLNA, FOXO1, FOXO3, IRS1, NFKBIA, PGK1, and STAT3) were selected by intersecting the top-ranked genes identified through five centrality algorithms (MCC, Degree, Betweenness, DMNC, and Closeness) in Cytoscape’s CytoHubba plugin. **(C)** Heatmap displaying the expression profiles of the selected biomarker genes across seven independent microarray training datasets. Color gradients represent relative gene expression levels across samples. The consistency in expression patterns supports their relevance across cohorts.

To uncover the functional roles and providing insights into biological processes, we conducted PPI network analysis of the 99 common significantly ranked genes using STRING (Figure 2A) and identified hub genes using the cytoHubba plugin in Cytoscape (Shannon et al., 2003; Chin et al., 2014). We next selected the top 20 genes using the MCC, Degree, Betweenness, DMNC, and Closeness methods (see ‘Materials and Methods’ section). Using a Venn diagram, we found that BCL6, DDIT4, FLNA, FOXO1, FOXO3, IRS1, NFKBIA, PGK1, and STAT3 were consistently identified by all five algorithms (Figure 2B). Analysis of the expression levels of these nine genes across five osteoporosis datasets and two sarcopenia datasets revealed that, with few exceptions, these genes generally exhibited moderate differential expression between disease and healthy samples (Figure 2C). This suggests that further pathway analysis is warranted.

### 3.2 Network and enrichment analysis

**FIGURE 3:**
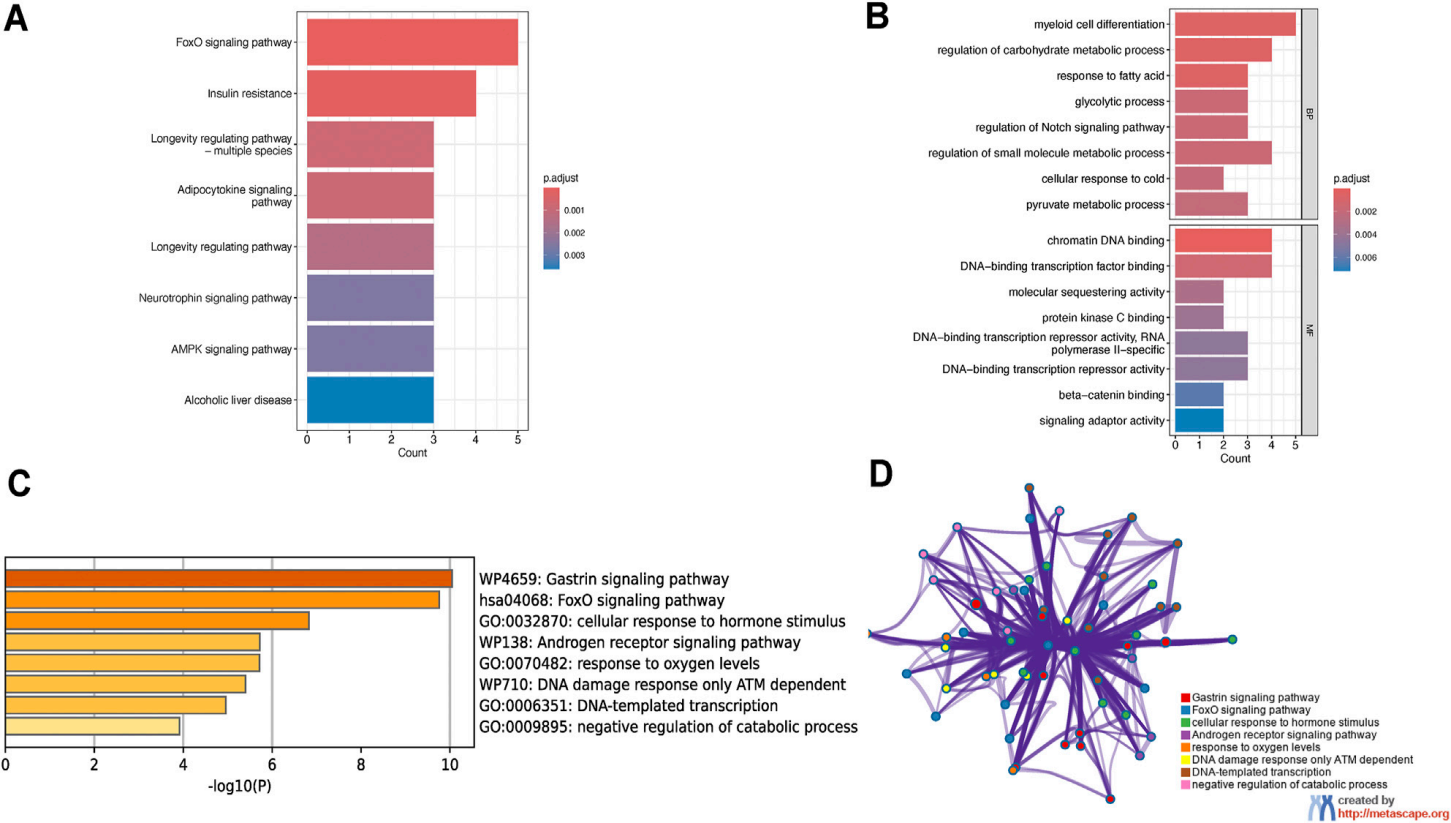
Functional enrichment analysis of osteoporosis-sarcopenia biomarker genes. **(A)** KEGG pathway analysis shows top enriched pathways including FoxO signaling, insulin resistance and longevity regulation. Pathways are ranked by significance and gene count, with FoxO signaling being most prominent. **(B)** GO analysis reveals key biological processes like myeloid cell differentiation and molecular functions including chromatin DNA binding, all statistically significant. **(C)** Metascape enrichment displays top terms colored by significance level, with darkest yellow indicating highest significance. **(D)** Metascape network visualization groups related terms into functional modules, demonstrating connections between pathways like FoxO signaling and hormone response.

To explore the potential biological mechanisms underlying these observations, we performed KEGG pathway analysis. In addition to providing curated biological pathways that map molecular interactions, reactions, and relationships, the analysis revealed significant enrichment of these nine genes in eight pathways, including the FoxO signaling pathway, insulin resistance pathway, and longevity regulating pathway (Figure 3A). Gene ontology analysis revealed significant enrichment in biological processes such as myeloid cell differentiation, regulation of carbohydrate metabolic processes, and regulation of small molecule metabolic processes. Additionally, these genes were significantly enriched in molecular functions like chromatin DNA binding and DNA-binding transcription factor binding (Figure 3B). To enhance the reliability of our results, we also performed enrichment analysis using Metascape. This analysis revealed significant enrichment in pathways such as the gastrin signaling pathway, FoxO signaling pathway, cellular response to hormone stimulus, androgen receptor signaling pathway, DNA damage response only ATM dependent, DNA-templated transcription, and negative regulation of catabolic processes (Figures 3C,D). Notably, the FoxO signaling pathway was significantly enriched in both the KEGG and Metascape analyses.

### 3.3 Machine learning models and SHAP analysis

For precise molecular diagnosis of osteoporosis and sarcopenia, as well as validation of these identified hub genes as common biomarkers for both diseases, we trained the machine learning model using GSE35959, GSE62402, GSE13850, GSE56814, GSE56815, GSE1428, and GSE136344 datasets. However, due to the absence of gene expression data for FOXO3 in certain datasets (missing in GSE62402 and GSE56814), we used the expression levels of the remaining eight consensus genes—BCL6, DDIT4, FLNA, FOXO1, IRS1, NFKBIA, PGK1, and STAT3—as features for the machine learning model framework. Before training the model, we standardized the data within each dataset to ensure comparability across different datasets. We used the ‘StandardScaler()’ function from Scikit-learn to normalize the samples. We used 80% of the data for training and reserved 20% for validation. We evaluated the model’s performance using 5-fold cross-validation.

To objectively assess model performance, we utilized the independent test datasets GSE7429 and GSE362, which contain data for osteoporosis and sarcopenia, respectively. The discriminative power of the identified biomarkers was first examined by evaluating each gene individually. Using their expression levels as the sole feature, we assessed their ability to distinguish between disease and healthy samples in the test datasets (Supplementary Figures S2, S3). The results indicated that none of the eight biomarker genes alone could provide robust classification performance for both diseases simultaneously.

**FIGURE 4:**
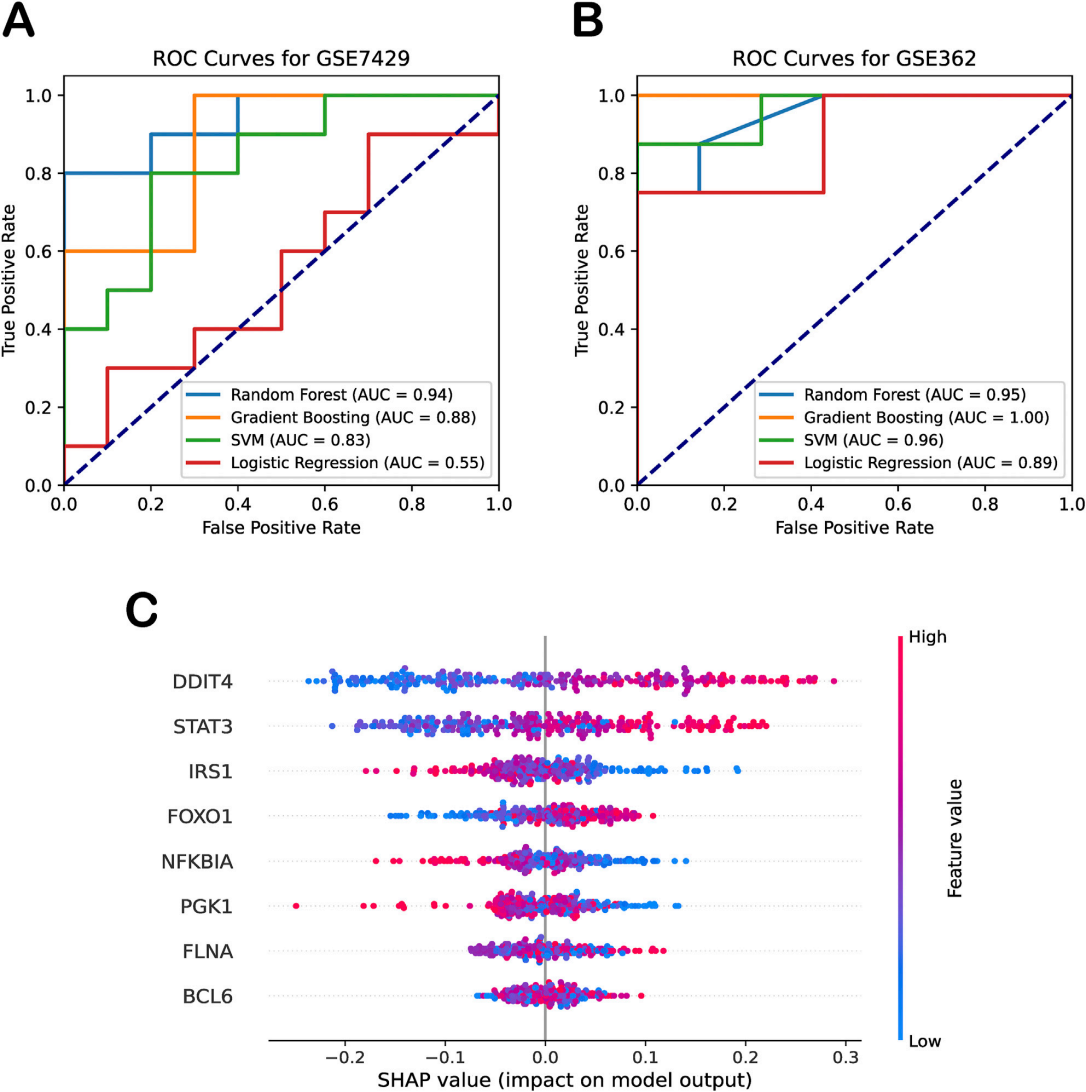
Diagnostic performance and interpretability of machine learning models. **(A)** ROC curves for four machine learning models on the independent osteoporosis test dataset (GSE7429). **(B)** ROC curves for the same models on the independent sarcopenia test dataset (GSE362). **(C)** SHAP beeswarm plot summarizing feature contributions to random forest model predictions.

We further expanded our analysis by employing logistic regression—a simple linear model—as a baseline approach to evaluate the classification performance of the biomarker genes without relying on complex machine learning techniques. As shown in Figures 4A,B, the independent test datasets showed that this baseline model achieved an AUC of 0.58 for osteoporosis (GSE7429) and 0.91 for sarcopenia (GSE362), suggesting that, while the biomarker genes collectively exhibit moderate yet imbalanced predictive power, more advanced models may improve classification performance. Building on this, we applied random forest, gradient boosting, and SVM models to fully exploit the predictive potential of these biomarkers. The results demonstrated that integrating these genes into advanced machine learning frameworks effectively distinguished diseases from normal control groups (Figures 4A,B). The gradient boosting model achieved AUCs of 0.88 and 1.00 for osteoporosis and sarcopenia, respectively, while the SVM model attained AUCs of 0.83 and 0.96. Notably, the random forest model provided the most balanced and robust performance, with AUCs of 0.94 and 0.95 across the two independent datasets. Given these results, we selected random forest as the default model for our framework.

To quantify the contributions of biomarker genes in our random forest model, we employed SHAP analysis to explain the model’s performance. SHAP beeswarm summary plot presented a dense summary of how the biomarker genes in the dataset influenced the output of the random forest model. Specifically, the SHAP values (on the x-axis) illustrate the impact of the biomarker genes (on the y-axis) on the random forest model’s predictions, with each dot representing the contribution of the gene’s expression level in a specific sample to the model’s classification decision. This provided a measure of each feature’s contribution to the model’s output, with higher means absolute SHAP values indicating greater influence. As shown in Figure 4C, SHAP analysis revealed that DDIT4, STAT3, and FOXO1 had significant impact on the model. Their effects were positive, meaning that higher expression levels contributed to the classification of disease, whereas lower expression levels favored the classification of healthy samples. In contrast, IRS1 and PGK1 exhibited an opposite trend to some extent. Moreover, we observed that the SHAP analysis using the training dataset yielded results consistent with our validation findings on the independent test set. These results further support the robustness and reliability of our machine learning model in identifying key biomarkers for osteoporosis and sarcopenia.

### 3.4 Computational and experimental validation

**FIGURE 5:**
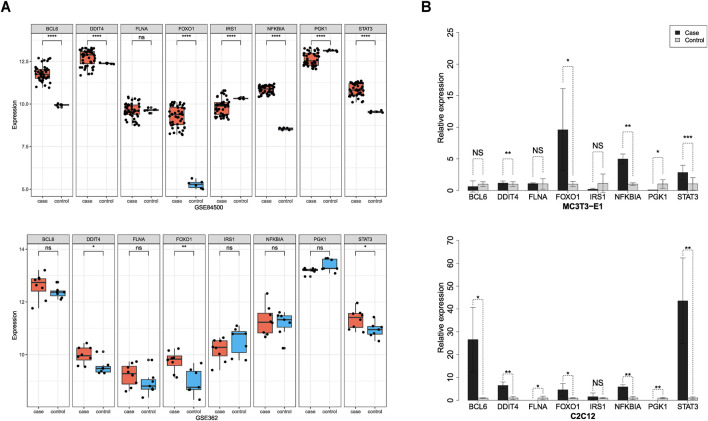
Computational and experimental validation of the biomarker genes. **(A)** Computational validation using independent test datasets. DDIT4, FOXO1, and STAT3 were significantly differentially expressed in both the osteoporosis (GSE84500) and sarcopenia (GSE362). **(B)** Experimental validation using *in vitro* models. RT-qPCR analysis confirmed that DDIT4, FOXO1, and STAT3 were significantly upregulated in both osteoporosis and sarcopenia *in vitro* models.

To verify the accuracy of the biomarker genes identified by our machine learning model, we conducted both computational and experimental validations. For computational validation, we assessed the significance of the eight consensus biomarker genes across independent datasets. Differential expression analysis in osteoporosis and sarcopenia datasets revealed that seven biomarkers—BCL6, DDIT4, FOXO1, IRS1, NFKBIA, PGK1, and STAT3—were differentially expressed in the independent osteoporosis dataset GSE84500. Meanwhile, DDIT4, FOXO1, and STAT3 exhibited significant differential expressions in the sarcopenia dataset GSE362 (Figure 5A).

To experimentally validate the expression patterns of eight consensus biomarker genes, we established *in vitro* models of osteoporosis and sarcopenia using MC3T3-E1 and C2C12 cell lines, respectively. The osteoporosis model exhibited characteristic alterations in key regulatory factors of osteogenic differentiation (RUNX2), OPG, and RANKL, while the sarcopenia model demonstrated specific expression changes in MYOG and ubiquitin ligases Atrogin-1 and MuRF1. Significant differential expressions of these molecules confirmed the validity of model establishment (Supplementary Figure S4). Using the established models, we performed RT-qPCR to quantify gene expression levels in cells affected by osteoporosis and sarcopenia. Results revealed significant differential expressions of DDIT4, FOXO1, NFKBIA, PGK1, and STAT3 in the osteoporosis model. Correspondingly, the sarcopenia model showed marked differential expressions of BCL6, DDIT4, FLNA, FOXO1, NFKBIA, PGK1, and STAT3 (Figure 5B).

Overall, our findings demonstrate the consistency between computational validation and RT-qPCR experimental results. Our proposed systems biology framework and machine learning model can effectively identify reliable biomarkers. By combining computational and experimental validations, we confirmed that at least DDIT4, FOXO1, and STAT3 were significantly upregulated in both osteoporosis and sarcopenia, providing strong support for precise disease diagnosis, further exploration of comorbidity mechanisms, and potential therapeutic interventions. Moreover, these findings highlight the complexity of osteoporosis and sarcopenia as age-related diseases influenced by multiple genes, with pathway interactions and protein networks playing critical roles in disease progression.

## 4 Discussion

Emerging evidence suggests a close relationship between osteoporosis and sarcopenia; however, the molecular mechanisms linking them remain poorly defined. To address this gap, we applied a systems biology framework to analyze transcriptomic data from both diseases. Our integrative analysis identified key genes—BCL6, DDIT4, FLNA, FOXO1, IRS1, NFKBIA, PGK1, and STAT3—potentially involved in their pathogenesis.

Functional enrichment analysis revealed that the FoxO signaling pathway was significantly and consistently enriched across both KEGG and Metascape platforms, highlighting its potential as a central regulatory axis linking osteoporosis and sarcopenia. FoxO transcription factors, particularly FOXO1, are key regulators of cellular homeostasis and longevity. In skeletal muscle, FOXO1 upregulates atrophy-related genes such as Atrogin1 and MuRF1, thereby promoting muscle protein degradation and contributing to sarcopenia (Sandri et al., 2004; O’Neill et al., 2018). In bone tissue, FOXO1 also plays essential roles in osteoblast differentiation, redox balance, and bone remodeling, with its dysregulation associated with reduced bone mass and impaired repair (Siqueira et al., 2011). Beyond FOXO1 itself, DDIT4 emerged as another biomarker tightly linked to the FoxO pathway. DDIT4 is a stress-responsive gene that negatively regulates the mTOR pathway, thereby indirectly promoting FoxO transcriptional activity under conditions of oxidative or metabolic stress (Gharibi et al., 2016; Altab et al., 2025). This link may explain how systemic stress contributes to degeneration in both muscle and bone tissues. Additionally, STAT3, while not a canonical component of the FoxO pathway, interacts with it via shared downstream targets and stress response circuits. STAT3 is involved in IL-6–mediated inflammatory signaling, which has been implicated in both muscle wasting and bone resorption. Notably, several studies suggest crosstalk between STAT3 and FoxO proteins in regulating oxidative stress and cell survival, pointing toward their convergence in age-related musculoskeletal disorders (Levy and Loomis, 2007; Milner et al., 2015; Guadagnin et al., 2018). Collectively, these findings underscore that FoxO signaling does not act in isolation but rather serves as a molecular hub, integrating multiple upstream regulators (e.g., DDIT4) and intersecting with inflammatory pathways (e.g., STAT3), thereby contributing to the shared pathogenesis of osteoporosis and sarcopenia. This not only provides a mechanistic explanation for the co-occurrence of these conditions but also suggests the FoxO pathway as a promising target for therapeutic intervention.

To validate these findings, we examined gene expression across independent datasets and observed consistent differential expression of DDIT4, FOXO1, and STAT3 in both osteoporosis and sarcopenia cohorts, confirming their relevance as shared biomarkers. This systems-level insight not only uncovers potential mechanistic links between the two diseases but also informs future therapeutic targeting strategies. Moreover, we developed a machine learning-based classification framework leveraging these biomarkers, which achieved high accuracy in distinguishing between the two conditions. Finally, our *in vitro* experiments further validated the consistency of these findings, demonstrating that the integration of computational biology and experimental validation provides a robust approach for early and precise diagnosis of age-related musculoskeletal diseases.

Although our study contributes to establishing the molecular connection between osteoporosis and sarcopenia, it also has several limitations. First, due to current data constraints, we were unable to include single-cell or bulk RNA-seq transcriptomic analyses to explore the cellular composition and origins of the observed gene expression changes. A more fine-grained, cell-type-specific approach could offer deeper mechanistic insights into the pathogenesis of osteoporosis and sarcopenia. Collecting patient-derived samples or obtaining relevant single-cell data for analysis will be a key priority in our future work. Second, although we identified several key genes potentially involved in both diseases, the exact molecular roles of these genes in the development and progression of osteoporosis and sarcopenia remain to be fully clarified. Future research should include more in-depth molecular biology experiments—such as gene editing or pathway perturbation studies—to better elucidate their mechanistic functions in this comorbidity. Third, our validation strategy combined computational analysis with *in vitro* modeling and RT-qPCR experiments using mouse cell lines. While MC3T3-E1 and C2C12 cells have been widely used in bone and muscle research and share high genetic conservation with human tissues, they cannot fully replicate the complexity of human disease pathology. Although *in vitro* systems offer the advantages of experimental control and reproducibility, the absence of animal models or clinical samples limits the translational applicability of our findings. Future studies incorporating patient-derived tissues or *in vivo* animal models will be essential to validate the clinical relevance of the identified biomarkers. Despite these limitations, we believe our study contributes to a better understanding of the shared molecular basis of osteoporosis and sarcopenia and may inform future research aimed at biomarker-driven diagnostics and the development of targeted therapeutic strategies.

## Data Availability

The datasets analyzed for this study can be found in the Gene Expression Omnibus database repository (See Supplementary Table S1 for the detailed accession numbers). Our proposed framework is implemented in R and Python and is freely available from the GitHub repository at https://github.com/tanlaboratory/osteoporosis_sarcopenia_analysis.
