# Supplementary material for: Systems biology and experimental validation indicate DDIT4, FOXO1, and STAT3 as shared key genes linking osteoporosis and sarcopenia
Source: Front Genet. 2025 Nov 6;16:1630705. doi: 10.3389/fgene.2025.1630705 (PMC12631428; doi:10.3389/fgene.2025.1630705)
Supplement: Supplementary file 1 [file DataSheet1.docx]

Supplementary Material

1. **Supplementary Figures and Tables**


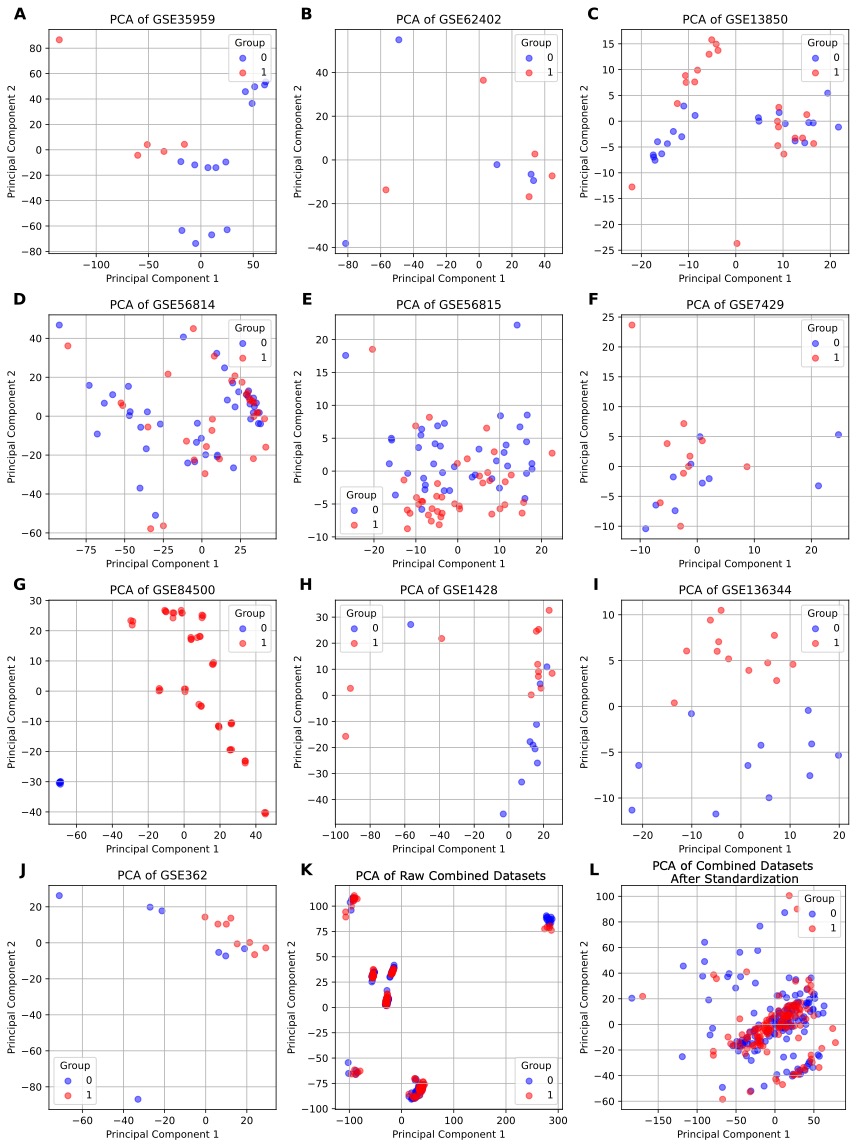


Supplementary Figure S1. Principal component analysis (PCA) plots for individual datasets (A to J) and the combined datasets (K and L). (A to J) PCA was conducted separately for each of the 10 datasets included in this study, following differential gene expression analysis. The results indicate that, in most datasets, cases (red) and controls (blue) are distinctly separated, highlighting significant gene expression differences between disease and healthy samples. This supports the approach of identifying differentially expressed genes (DEGs) within each dataset as a strong foundation for disease classification. (K) The PCA plot of the combined unnormalized dataset shows that case and control samples from each individual dataset form separate clusters, indicating notable within-dataset homogeneity but strong heterogeneity across datasets. This validates our method of first identifying DEGs within each dataset and then integrating them using the RRA approach to reduce dataset-specific noise. (L) The PCA plot for the standardized combined dataset shows a lack of clear linear separation between disease and control groups, underscoring the need for advanced machine learning models to accurately distinguish between the two conditions.

Supplementary Figure S2. Performance of each biomarker gene on the GSE7429 dataset. The classification performance of each biomarker gene was assessed by using its expression level as the sole feature for distinguishing between disease and healthy samples in the osteoporosis test dataset (GSE7429).

Supplementary Figure S3. Performance of each biomarker gene on the GSE362 dataset. The classification performance of each biomarker gene was assessed by using its expression level as the sole feature for distinguishing between disease and healthy samples in the sarcopenia test dataset (GSE362).

| 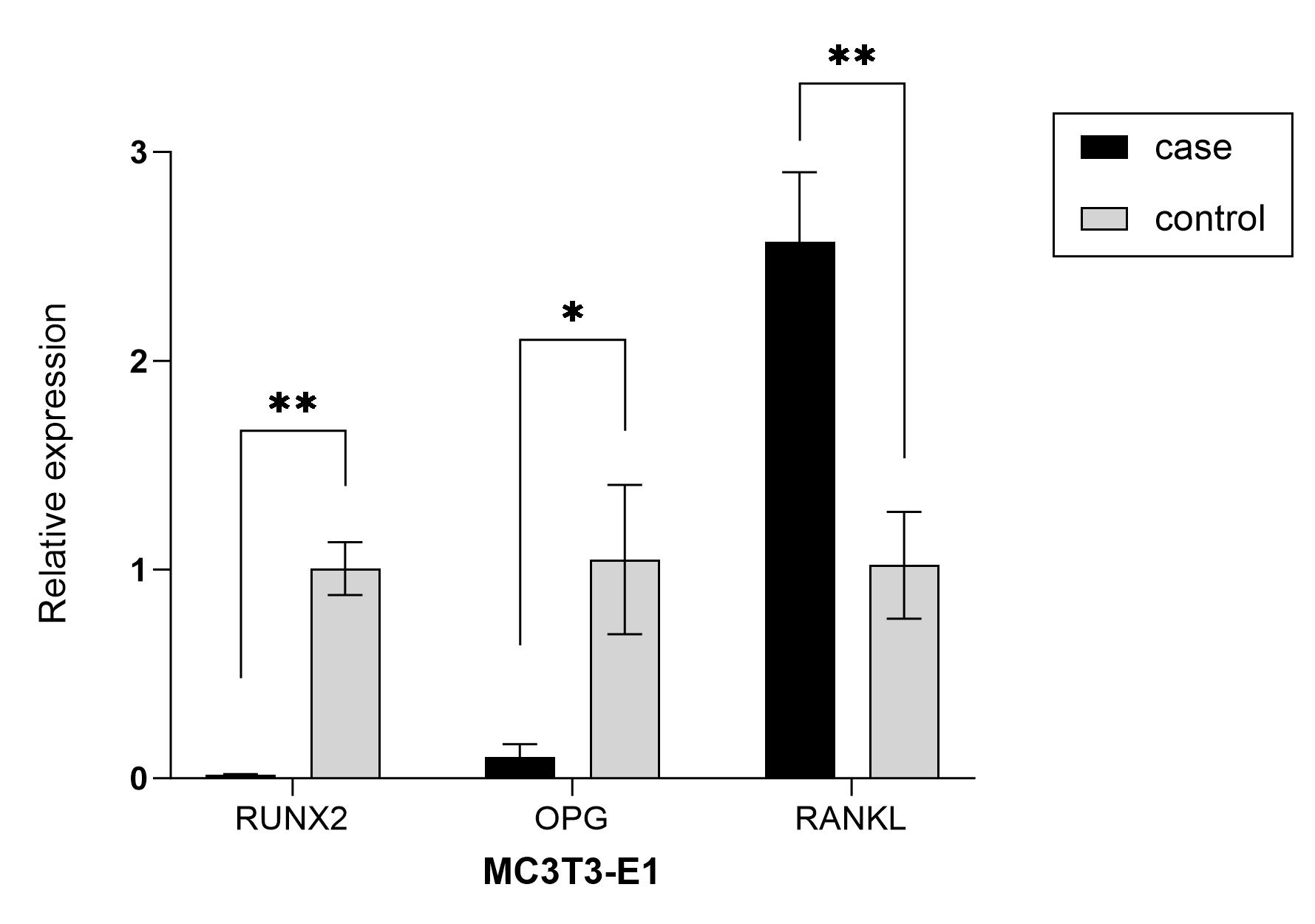 | 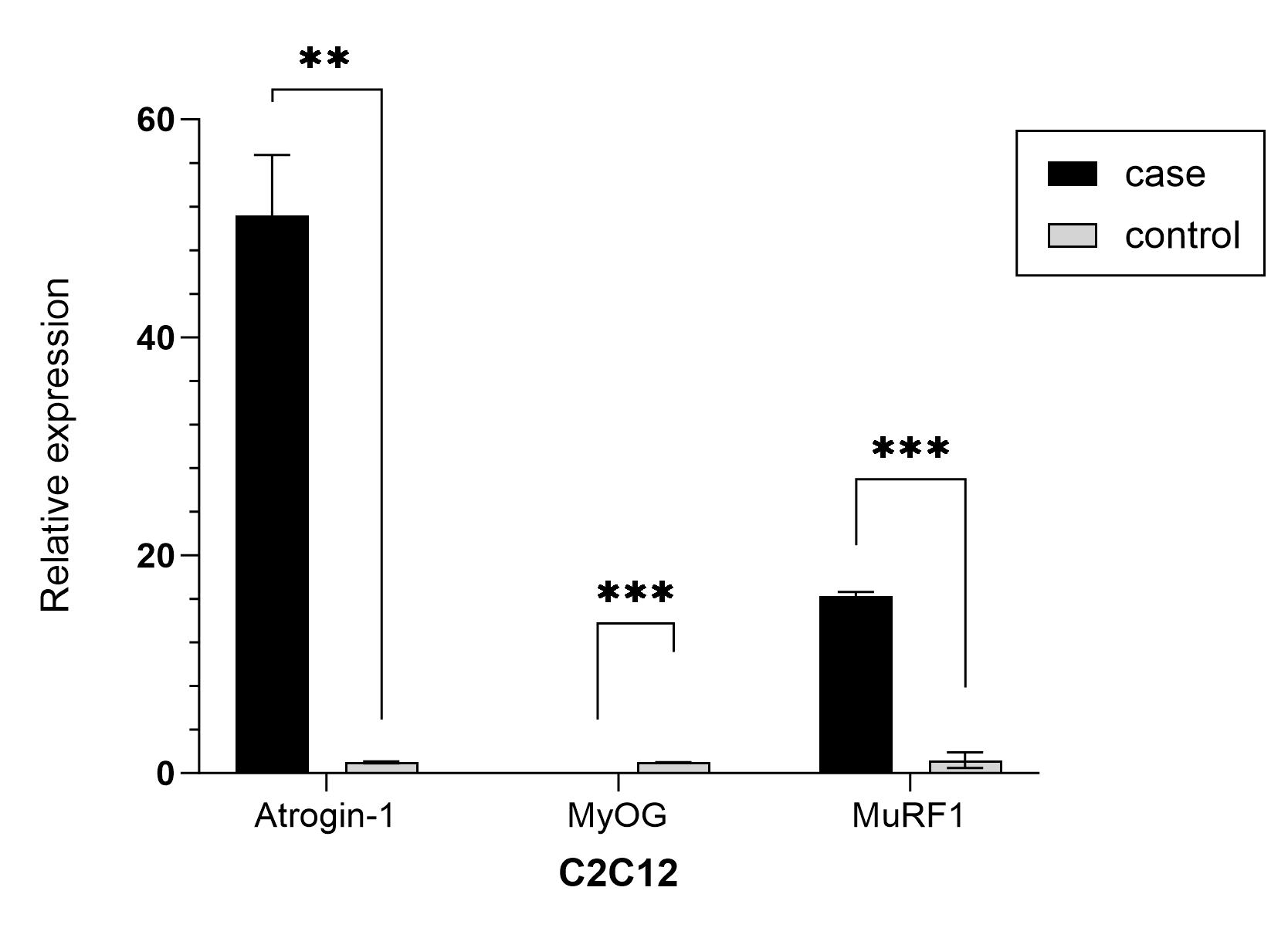 |
| --- | --- |

Supplementary Figure S4. Experimental verification of markers in established osteogenic and sarcopenia models to confirm model reliability. Experimental validation showed differential expressions of the key markers RUNX2, OPG and RANKL in an osteoporosis model (MC3T3-E1). In the sarcopenia model (C2C12), there were differences in the expression of the key biomarkers MYOG, Atrogin-1 and MuRF1.

Supplementary Table S1. Microarray datasets with cell source annotations obtained from the Gene expression omnibus repository and their classification for the machine learning model

| Dataset | Disease | Sample size | Dataset classification | Cell source |
| --- | --- | --- | --- | --- |
| GSE13850 | Osteoporosis | 20 cases, 20 controls | Training set | B cells (from whole blood) |
| GSE35959 | Osteoporosis | 5 cases, 14 controls | Training set | Mesenchymal stem cells |
| GSE62402 | Osteoporosis | 5 cases, 5 controls | Training set | Circulating monocytes |
| GSE56814 | Osteoporosis | 31 cases, 42 controls | Training set | Circulating monocytes |
| GSE56815 | Osteoporosis | 40 cases, 40 controls | Training set | Circulating monocytes |
| GSE1428 | Sarcopenia | 12 cases, 10 controls | Training set | Vastus lateralis muscle |
| GSE136344 | Sarcopenia | 12 cases, 11 controls | Training set | Vastus lateralis muscle |
| GSE7429 | Osteoporosis | 10 cases, 10 controls | Testing set | B cells (from whole blood) |
| GSE84500 | Osteoporosis | 48 cases, 6 controls | Testing set | Mesenchymal stem cells |
| GSE362 | Sarcopenia | 8 cases, 7 controls | Testing set | Vastus lateralis muscle |
